# Study protocol: understanding pain after dental procedures, an observational study within the National Dental PBRN

**DOI:** 10.1186/s12903-022-02573-9

**Published:** 2022-12-09

**Authors:** Elisabeth Kalenderian, Joel White, Alfa-Ibrahim Yansane, Janelle Urata, David Holmes, Kimberly Funkhouser, Rahma Mungia, Jin Xiao, Cindy Rauschenberger, Ana Ibarra-Noriega, Duong Tran, D. Brad Rindal, Heiko Spallek, Muhammad Walji

**Affiliations:** 1 Academic Center for Dentistry at Amsterdam (ACTA), Gustav Mahlerlaan 3004, Room 6N-09, 1081 Amsterdam, LA The Netherlands; 2 University of California San Francisco, School of Dentistry, 600 Parnassus Avenue, San Francisco, CA USA; 3 Harvard School of Dental Medicine, Boston, MA USA; 4 University of Pretoria, School of Dentistry, Pretoria, South Africa; 5 FollowApp.Care, London, England; 6 Private Dental Practice, Periodontics and Implant Dentistry, 19 Wimpole St, W1G 8GE London, London UK; 7 Kaiser Permanente Center for Health Research, 3800 N. Interstate Avenue, Portland, OR 97227-1098 USA; 8 UTHealth San Antonio, School of Dentistry, San Antonio, TX USA; 9 Department of Dentsitry, Eastman Institute for Oral Health, National Dental PBRN, 625 Elmwood Ave, Box 683, Rochester, NY 14620 USA; 10 The University of Texas Health Science Center at Houston, School of Dentistry, Diagnostic and Biomedical Sciences, Research Office, 7500 Cambridge Street, room 4334, Houston, TX 77054 USA; 11 HealthPartners Institute for Education and Research, 8170 33rd Avenue South, P.O. Box 1524, MS 23301A, Bloomington, MN 55440-1524 USA; 12 University of Sydney, School of Dentistry, 2 Chalmers St., Surry Hills, Sydney, NSW 2010 Australia

**Keywords:** Pain, Dentistry, Patient-reported outcomes, Mobile health technology, National Dental Practice-Based Research Network, Observational study

## Abstract

**Background:**

Patient-reported outcome measures provide an essential perspective on the quality of health care provided. However, how data are collected, how providers value and make sense of the data, and, ultimately, use the data to create meaningful impact all influence the success of using patient-reported outcomes.

**Objectives:**

The primary objective is to assess post-operative pain experiences by dental procedure type through 21 days post-procedure as reported by patients following dental procedures and assess patients’ satisfaction with pain management following dental surgical procedures. Secondary objectives are to: 1) assess post-operative pain management strategies 1 week following dental surgical procedures, as recommended by practitioners and reported by patients, and 2) evaluate practitioner and patient acceptance of the FollowApp.Care post visit patient monitoring technology (FollowApp.Care). We will evaluate FollowApp.Care usage, perceived usefulness, ease of use, and impact on clinical workload.

**Design and methods:**

We describe the protocol for an observational study involving the use of the FollowApp.Care platform, an innovative mobile application that collects dental patients’ assessments of their post-operative symptoms (e.g., pain). The study will be conducted in collaboration with the National Dental Practice-based Research Network, a collective Network of dental practices that include private and group practices, public health clinics, community health centers and Federal Qualified Health Centers, academic institutional settings, and special patient populations. We will recruit a minimum of 150 and up to 215 dental providers and up to 3147 patients who will receive push notifications through text messages FollowApp.Care on their mobile phones at designated time intervals following dental procedures. This innovative approach of implementing an existing and tested mobile health system technology into the real-world dental office setting will actively track pain and other complications following dental procedures. Through patients’ use of their mobile phones, we expect to promptly and precisely identify specific pain levels and other issues after surgical dental procedures.

The study’s primary outcome will be the patients’ reported pain experiences. Secondary outcomes include pain management strategies and medications implemented by the patient and provider and perceptions of usefulness and ease of use by patients and providers.

**Supplementary Information:**

The online version contains supplementary material available at 10.1186/s12903-022-02573-9.

Contributions to the literature

- Patient self-report is a critical part of comprehensive pain assessment. By promptly collecting patient health assessments, we will offer practitioners an opportunity to assess the effectiveness of their treatment modalities to understand their patients’ experiences and health outcomes.
- In this study, dental practitioners can respond in real-time to patient-reported outcomes for post-operative pain management.
- Evidence suggests that mobile phones are an effective platform for assessing patients’ symptoms and symptom burden. In addition, mobile applications can be effective in collecting and utilizing patient-reported outcomes to tailor care, however, they are rarely used in dentistry.
- Knowledge of treatment recommendations and/or patient reports will provide useful information in the shaping of dental school curricula.

## Background

Existing means to address unexpected post-operative discomfort following a dental procedure include preemptively prescribing pain medication that might not be needed or having the patient call/visit the dental clinic before the regularly scheduled follow-up time period. Both approaches do not reflect patient-centered care and carry the risk of under/ overprescribing analgesics - including opioids. As the PEARL network concluded in its 2015 study examining dentists’ post-procedural prescriptions, “dentists tended to expect more pain than patients actually experienced; however, the choice of medication prescribed or recommended did not reflect the dentists’ expectations or the patients’ outcomes [1].” Oral health providers’ inability to monitor post-operative pain in real-time, and their desire to prevent unwanted, unscheduled visits, have driven them to heavily prescribe opioids in a failed attempt to satisfy patients’ short-term pain management [2–4].

Patient-reported outcomes (PROs) provide an essential perspective on the quality of health care and facilitate clinical decision-making. Electronic and paper-based administration of PROs has proven to be equally effective [5]. Data collection procedures, along with how providers value and make sense of the data and, ultimately, use it to create meaningful impact, all influence the success of using PROs [6]. PROs are widely used to inform individual care and manage the performance of healthcare [7]. However, assessing PROs in real-time as part of out-patient care is challenging. Studies on the use of PROs in medical health systems show that PROs must be easy (simple user-interface, convenient timing), fast (short questionnaire length and frequency), and relevant (inform clinical care). For providers, PROs should make care easier (reduce administrative burden), faster/better (improve quality of visit), and relevant (solve discipline-specific problems) [8]. Unfortunately, the use of PROs in dentistry is not widespread, thereby limiting the ability of dental providers to respond in real-time to post-operative pain management by unlocking the power of PROs.

Current applications of Health Information Technologies (HIT) in dentistry are limited to electronic health records (EHRs) in general [9, 10], using EHRs for non-clinical management purposes [10–13], and unconnected “gadgets”, such as CAD/CAM systems, intraoral cameras or ConeBeam CTs. However, growing evidence from our medical colleagues suggests that mobile phones are an effective platform for assessing patients’ symptoms, symptom burden, health status, health behaviors, and health-related quality of life [14–19]. For example, a study by Stein et al. explored the feasibility of using smartphone applications to triage dental emergencies [20]. Another study from the UK revealed that mobile applications motivated dental patients to maintain better oral hygiene [21]. With 85% of US adults owning a cellphone [22], and 67% of patients using their phones to search for health information [23], dental providers need to embrace HIT as a strategy for delivering quality care to improve the oral health their patients. This is especially important when interacting with low SES families as a significant proportion of them only connect to the Internet via smartphones [24].

While research on the effectiveness of text messages and mobile app-related inventions to improve the quality of care is still in its infancy, results from studies using other forms of HIT are promising. For instance, mPOWEr enables patients to provide feedback on the condition of their surgical incision site following discharge from the hospital, while other mobile apps that collect PRO data enable patients to monitor their chronic health conditions, such as diabetes [25, 26]. Additionally, participants in a chemotherapy treatment group using a web-based PRO questionnaire platform to report their symptoms had fewer hospitalizations, emergency room visits, and overall higher survival rates [27, 28]. Results of studies like these indicate that mobile apps could effectively collect and utilize PROs to tailor care [29], particularly in dentistry, where HIT is rarely used to collect PROs.

In our pilot study (AHRQ 1U18HS026135), we successfully explored how an innovative mobile health intervention (FollowApp.Care®) could optimize the quality of acute post-op dental pain management by collecting PRO data from patients after dental procedures. However, it also taught us a number of implementation barriers. Some of these barriers are not new to those collecting PRO data, including difficulties with optimizing workflows and minimizing logistical burdens, further exacerbated by the expanded precautionary settings during COVID; data management in light of staff turnover; and staff training and providers [30, 31]. Barriers specific to implementing dental PROs using mHealth included making sure that providers see and act upon alerts timely; a hybrid approach to recruitment of patients due to the COVID environment; inaccurate phone numbers, and phone carrier contractual issues that prevented text messaging.

These barriers can substantially hamper the implementation of PROs by the general dentist, to the detriment of advancing patient-centered care in the dental arena. In the POPS (**P**ost-**O**perative **P**ain **S**tudy) trial (UH3DE029158, Understanding Pain after Dental Procedures), we seek to address the gap in knowledge by implementing an existing mHealth technology (FollowApp.Care) in the dental office setting. Through the FollowApp.Care platform, we will actively track pain and other complications following dental procedures. We expect to identify specific pain experiences associated with surgical dental procedures promptly and precisely. We will also collect information about pain management strategies and medications recommended by the practitioner and utilized by the patient. The results of this study will provide insight into pain and other postoperative complications experienced by patients through approximately 3 weeks following common dental procedures. We will also be able to gauge to what extent practitioners are interested in adopting mHealth technology. We will also learn how patients like to be engaged with text messaging to report post-operative pain and complications.

This study was funded as a result of RFA-DE-19-006, National Dental Practice-Based Research Network: Clinical Trial or Observational Study Planning and Implementation Cooperative Agreement. This study was funded as part of the National Institute of Dental and Craniofacial Research’s support for research conducted within the National Dental Practice-Based Research Network (PBRN; “Network”) through the UG3/UH3 mechanism. The study was approved by the central IRB (cIRB) of the University of Alabama at Birmingham (UAB), which manages all clinical studies for the Network. All participating entities’ IRB ceded to UAB’s cIRB as required.

A populated checklist from the relevant reporting guideline(s) appropriate for this cohort study design is attached.

## Objectives

### Primary objective is

To assess post-operative pain experiences by dental procedure type through 21 days post-procedure as reported by patients following study-eligible dental procedures and assess patients’ satisfaction with pain management following dental surgical procedures.

### Secondary objectives are

1. Assess post-operative pain management strategies at 1 week following dental surgical procedures, as recommended by practitioners, and reported by patients.
2. Evaluate practitioner and patient acceptance of FollowApp.Care. We will evaluate FollowApp.Care usage, perceived usefulness, ease of use, and impact on clinical workload.

## Hypotheses

The study includes the following hypotheses:

### Hypothesis 1

There is a significant difference in pain intensity measured over time and the dental procedure groupings after adjusting for pain management strategies and other complications. More specifically, are their significant variations in pain intensity due to different treatments after adjusting for:

- pain management strategy (e.g., pain medications used, adherence to pain management strategy, the usage of non-medicine methods for pain)
- other complications

### Hypothesis 2

There is a significant difference in pain interference measured over time and the dental procedure groupings, after adjusting for pain management strategies and other complications. More specifically, are their significant variations in pain interference due to different treatments after adjusting for:

- pain management strategy (e.g., pain medications used, adherence to pain management strategy, the usage of non-medicine methods for pain)
- other complications

### Hypothesis 3

There is a significant difference in patient satisfaction measured at the end of the 7-day period and dental procedure groupings, after adjusting for pain management strategies and other complications. More specifically, are their significant variations in patient satisfaction due to different treatments after adjusting for:

- pain management strategy (e.g., pain medications used, adherence to pain management strategy, the usage of non-medicine methods for pain)
- other complications.

### Hypothesis 4

Reported technology acceptance metrics; performance expectancy (PE), effort expectancy (EE), social influence (SI), and behavioral intention (BI) will be consistent with high acceptance of the FollowApp.Care platform.

## Design and methods

### Study design

POPS is a longitudinal, prospective cohort study that will be conducted with practitioners and their patients undergoing potentially painful dental procedures. Data about baseline pain intensity and patient demographics will be collected prior to the procedure. The patients will receive push notifications through text messages via the FollowApp.Care platform on their mobile devices which asks them to comprehensively record their pain experience at designated time intervals on days 1, 3, 5, 7, 14, and 21 following their procedure. At the end of the 1st week (day 7), they will be asked to report their satisfaction with their post-operative pain management. On Day 23, after the procedure, they will be requested to complete a questionnaire to measure the platform’s usefulness and ease of use. All participating practitioners will be invited to complete a questionnaire to assess the platform’s perceived usefulness and ease of use. A subgroup of practitioners will also be invited to participate in debriefing/interviews to qualitatively evaluate their experiences with using FollowApp.Care for managing their patients’ post-op pain.

### Study sites

The study is being executed in the National Dental PBRN, a collective network of dental practices that includes private and group practices, public health clinics, community health centers and Federal Qualified Health Centers (FQHCs), academic institutional settings, and special patient population [32]. Since its inception in 2005, the Network has matured into a national network with coverage in urban and rural areas and participation by general dentists, dental specialists, and dental hygienists [33]. The Network is interested in increasing the study and use of HIT to collect PROs within its clinics. It is highly productive, with 19,827 practitioners participating in one or more Network studies [33], and 762 practitioners actively being part of at least one clinical study [34, 35]. The Network has been involved in 58 studies. Practice-based research promotes not only the collection of data at the provider and patient levels but also allows the gathering of patient perspectives before, during, or after the visit. The Network’s infrastructure has matured through various management initiatives into a nimble yet continuous, quality-improvement conscientious network [36]. It is rapidly moving toward achieving the vision of a Learning Health System [37]. Hence, this environment is ideal for developing a practical, focused post-operative pain management study. The six Network geographic regions in the US will be informed of the study protocol and opportunity to participate. We anticipate that all Network practitioners stratified by state, urban/rural, and practice size, will be eligible to participate; between 150 to 215 Network practitioners will be recruited.

### Study population and eligibility criteria

The study participants include Network dental practitioners who perform endodontic, periodontal, oral surgery, and/or implant dental surgical procedures and their patients. Eligibility criteria for practitioners include: 1) Be a Network practitioner deemed as study ready, 2) Be a dentist who performs at least one of the identified Code on Dental Procedures and Nomenclature (CDT Code) [38] procedures per week (Endo CDT codes D3000-D3999, Perio CDT codes D4200-D4299 and D4341 and D4342, Oral Surgery CDT codes D7000-D7999 excluding D7287, D7288, D7880, D7881, D7899, D7921, 12 CDT codes from Implant Services (D6000-D6199): D6010, D6011, D6012, D6013, D6040, D6050, D6100, D6101, D6102, D6103, D6104, D6081) and has access and willingness to use the platform through an internet browser using a smartphone, tablet, computer, or laptop for patient care purposes. Eligibility criteria for patients include: 1) Are willing and able to comply with all study procedures; 2) Planning to undergo one or more of the aforementioned surgical procedures; 3) Have access to and willingness to use their smartphone for study purposes, and 4) Have not already participated in the study previously. Each participating practitioner will be asked to recruit a minimum target of approximately three consenting patients per month, although recruitment targets will be further refined once the number of enrolled practitioners is clear. Practitioners may enroll patients at a rate and consecutive process (i.e., 1 day per week, 1 week per month, etc.) that best suits their practice situation.

### Study outcome measures

This study has two primary outcomes, which reflect 1) pain experience and 2) patient pain management satisfaction. *Pain experience* refers to both pain intensity and pain interference. *Pain intensity* is an assessment of how much a patient hurts. The response categories range from “No pain” to “Very severe” on a 10-point Likert scale [39]. Pain intensity is a relatively homogeneous dimension, and most measures of pain intensity tend to be interchangeable. It was selected because of its relevance to dental patients and practitioners, applicability to all conditions, including acute postoperative dental pain for assessing symptoms, ease of administration, and the relative accuracy with which most adult patients gauge pain. *Pain interference* refers to the extent to which patients have experienced interferences that have prevented them from activities of daily living (ADL), falling asleep, and staying asleep. The response categories range from “Does not interfere” to “Completely interferes” on a 10-point Likert scale. *Patient pain management satisfaction* refers to the level with which patients are satisfied with the overall pain management following their procedure and shared pain management strategies. The response categories range from “Extremely dissatisfied” to “Extremely satisfied” on a 10-point Likert scale. Additionally, it encompasses whether the patient participated in the decision-making regarding their treatment. The response categories range from “Not at All” to “Very Much So” on a 10-point Likert scale.

Secondary outcomes include 1) *Analgesic medications used by patients* that are reported through FollowApp.Care, frequency and dose prescribed captured through the eCRF completed by the practitioners; adherence to pain medications prescribed collected from the patient-reported data through FollowApp.Care, 2) *Concordance with pain management strategy* as recommended by the practitioner (reported on the eCRF) and reported by the patient. The pain management strategy of each practitioner will be captured on the eCRF, and the mHealth questionnaire will capture the corresponding patient adherence. 3) *Other pain management strategies* used by patients (post-op pain management strategies including usage of other pain management strategies, such as non-medicine methods for pain relief), 4) *Practitioner acceptance* of the platform (usage, perceived usefulness, ease of use, and impact on clinical workload), 5) *Patient perceived usability* with the platform (usefulness and ease of use of the platform), and 6) *Other complications* related to immediate postoperative complications (bleeding and increased swelling).

Other covariates are *Baseline pain intensity, Patient demographic* variables, *Type of procedure* (CDT), P*ractitioner demographic* variables, *Practitioner specialty, Practitioner years in practice (*the year of graduation from dental school), and Practitioner assessment of *expected pain intensity.*

### FollowApp.Care platform

FollowApp.Care is an existing communications platform that collects patient-generated health data prior to or after a procedure in order to inform treatment care decisions, drive quality, and generate actionable performance reports. This patient-monitoring mobile phone platform has the ability to link with EHR systems without requiring an application program interface (API) and has already been linked to EHRs in the US as well as in Europe and Australia. FollowApp.Care can be deployed through any text message-enabled phone and configured to deliver language translations (e.g., Spanish) and generate aggregate reports at the patient, provider, practice, and organizational levels. FollowApp.Care meets all HIPAA requirements. Data are stored in a self-operated data center and is encrypted in transit and at rest. Different permission levels within the system ensure different levels of accessibility of the data. Using the platform requires few steps (Table 1); its functionality can be summarized as follows:

- Level 1 Providers - able to see their own patient responses & profiles
- Level 2 Center Manager - able to see **all** patient responses and profiles within their assigned Center
- Level 3 Center SuperUser - able to see **all** patient responses and profiles of **all** Centers

**Table 1:** FollowApp.Care at a Glance

Step 1 - FollowApp.Care automatically sends patients a customized post-procedure text containing a web-link to a personalized cloud-based survey	
Step 2 - Patients respond to the survey	
Step 3 - Survey results are relayed back to the FollowApp.Care system in real-time	
Step 4 - Patients in need are identified based on customizable notification settings. The provider is notified by email or text if an action is required, an alert is created within the FollowApp.Care platform prioritized by severity	
Step 5 - The provider is able to respond to each case to ensure patient needs are met	

### Instruments for data collection

Specific instruments include:

1. mHealth questionnaires

The mHealth Questionnaire #1 includes questions about pre-procedural pain and patient demographics. The mHealth Questionnaire #2 includes questions about pain intensity and pain interference. Pain intensity questions are based on the PROMIS Item Bank v.1.0 – Pain Intensity Scale [39]. On days 1, 3, 5, 7, 14, and 21, patients are asked: “What is your level of pain right now?” On day 7, patients are also asked: “How intense was your pain at its worst since the procedure?” *Pain Interference* questions were taken from the validated Oral Health Impact Profile-14 (OHOP-14) [40] and the Revised American Pain Society Patient Outcome Questionnaire (APS-POQ-R) [41–43] questionnaires to focus attention on the small subset of activities (e.g., sleep) common to post-operative dental patients. If, on days 14 and 21, patients answer the pain intensity question (“What is your level of pain right now?”) as positive (> 0), they will also be asked the six pain interference questions. The mHealth Questionnaire #2 will also collect the following additional information: type and frequency of pain-relief medications taken and the presence of concurrent symptoms such as bleeding, visible swelling, suppuration (pus), and/or fever. The mHealth Questionnaire #3 includes questions from the 2nd mHealth questionnaire and questions about patient pain management satisfaction (see Additional file 1: Appendix 1).

2. System Usability Scale (SUS) questionnaire

*FollowApp.Care usability as perceived by patients will be measured* by the system usability scale (SUS) [44–46] questionnaire to measure the usefulness and ease of use of the FollowApp.Care platform. The validated SUS is a simple questionnaire that uses a ten-item attitude Likert scale, giving a global view of subjective usability assessments (see Additional file 2: Appendix 2).

3. Unified Theory of Acceptance and Use of Technology (UTAUT)

*Practitioner acceptance* with the FollowApp.Care platform will be measured by the Unified Theory of Acceptance and Use of Technology (UTAUT) [47] questionnaire to evaluate FollowApp.Care usage, perceived usefulness, ease of use, and impact on clinical workload. The UTAUT model measures the relationships between use intention and two independent constructs – performance expectancy and effort expectancy. The UTAUT model integrates eight major theories and has been tested and validated using large real-world data sets (see Additional file 3: Appendix 3).

4. electronic Case Report Form (eCRF)

Two eCRFs record practitioner information (practitioner characteristics) and patient information (patient demographics, diagnoses and procedures, pain levels, pain management plan, and complications) at Days 0 & 21 (see also Appendix 3).

5. Debriefing/interview

Telephone debriefing/interviews with purposefully selected practitioners will be conducted to qualitatively evaluate their experiences with using FollowApp.Care for managing their patients’ post-operative pain, including its impact on their clinic workload and workflow patterns, and satisfaction with the effectiveness of pain management.

### Study activities and data collection

Participating patients will be encouraged to complete the mHealth Questionnaire #1 prior to the procedure. Baseline pain intensity and patient demographic variables will be collected through the welcome message. *Type of procedure* (CDT) will be collected through the eCRF. *Practitioner demographic* variables, *specialty, and years in practice* will be collected through the practitioner’s enrollment questionnaire (EQ). This information is already available in the practitioner database, and enrolled practitioners will be asked to update their EQ before the study. *Practitioner years in practice* will be collected through the practitioner’s enrollment questionnaire (EQ).

Upon completing their procedures, all enrolled patients will receive post-operative instructions and guidance according to the provider’s standard practice. Additionally, all enrolled patients will receive guidance about FollowApp.Care. At the end of each day, the research staff will retrieve the new patient profiles from FollowApp.Care and check the patient contact information for completeness. The patients will receive text/email notifications at pre-determined time intervals (e.g., 9 am) on Days 1,3,5,7,14, and 21 and will be prompted to complete the corresponding mHealth Questionnaire (Table 2). An additional comment/chat feature will enable patients to securely communicate more information to their dental care team through the FollowApp.Care system, when needed. All patients will receive usual clinical care and can contact their provider/clinic by phone/in person if they so desire. Practitioner assessment of *expected pain intensity* will be collected through the eCRF.

**Table 2:** Overview of the instruments

Instrument	Completion Time	Content	Completed by
mHealth Questionnaire #1	Day 0 (pre-procedure)	- Pre-procedural pain - Patient demographics	Patients
mHealth Questionnaire #2	Days 1, 3, 5 and 14 and 21	- Pain intensity levels, medications used, bleeding, and swelling - Pain interference (if having pain on 14 and 21)	Patients
mHealth Questionnaire #3	Day 7	-Pain intensity levels, medications used, swelling -Pain interference -Satisfaction with pain management treatment	Patients
System Usability Scale (SUS) Questionnaire	Day 23	Patients’ perceptions of the usability of the FollowApp.Care user interface	Patients
UTAUT Questionnaire	After practitioner completes enrollment of all their patients	Explores four core constructs: performance expectancy, effort expectancy, social influence, and facilitating conditions as direct determinants of behavioral intention and behavior	Practitioners
electronic Case Report Form (eCRF)	Days 0 and 21	Two eCRFs: - Practitioner info (practitioner characteristics) - Patient info (diagnoses and procedures, pain levels, pain management plan, and complications)	Practitioners
Debriefing/interview	After practitioner completes enrollment of all their patients	Practitioners’ experiences with using FollowApp.Care for managing their patients’ post-op pain.	Practitioners

Patients will also be asked to evaluate the usability of the FollowApp.Care system by completing the 10-item, System Usability Scale survey and providers the UTAUT questionnaire. Once the patient component of the study has been concluded, we will conduct telephone interviews with providers to qualitatively evaluate their experiences with using FollowApp.Care for managing their patients’ post-operative pain, including its impact on their clinic workload, workflow patterns, and satisfaction with the effectiveness of pain management.

See Table 3 for an overview of study activities.

**Table 3:** Study activities

Task	Description of implementation/operationalization
1. Network Site Selection	We anticipate that all Network practitioners stratified by state, urban/rural, and practice size, will be eligible to participate. We will work with the Network coordinators to assure all sites are informed of the study protocol and the opportunity to participate.
2. Practitioner Recruitment and training	The Network site coordinators will use established protocols to recruit practitioners. The study team will work with the coordinators and the Network Communications Director to assure the dissemination of the study opportunity. The study team will provide the coordinators with all online training materials. Coordinators may decide to add an in-person training component. Study personnel will be available remotely via video conference/phone calls to support all training efforts.
3. Patient screening and enrollment	Each participating office will decide if practitioners or office staff will screen/enroll patients in the study. The office staff who enroll patients will need to have successfully completed appropriate training. Each participating office will maintain a log of screened and enrolled subjects. On a weekly basis, office staff will record their summary screening/enrollment information in the electronic data capture (EDC) system. Practitioners will enroll patients via a consecutive process that best suits their practice situation, i.e., they will approach any patient who may meet the inclusion criteria during the days and/or times when the office is participating in research activities.
4. FollowApp.Care platform activation	Enrolled practitioners will individually be able to request their FollowApp.Care notification preferences, for example, they can choose to receive notifications by email or text. Notifications will be sent to practitioners based on predefined thresholds defined in the manual of procedures (MOP).
5. Patient evaluation (Usability questionnaire)	Patients will complete a Usability survey (SUS) on day 23 using the FollowApp.Care platform.
6. Practitioner evaluation (debriefing/interview session and UTAUT questionnaire)	Once the patient component of the study has been concluded, we will administer the UTAUT Questionnaire to all participating practitioners to assess their perceived usefulness and perceived ease of use of FollowApp.Care as predictors of their usage behavior. Thereafter, we will conduct virtual debriefing/interviews with up to 45 purposefully selected practitioners to qualitatively evaluate their experiences with using FollowApp.Care for managing their patients’ post-op pain. These debriefing/interview discussions will be audiotaped and transcribed for analysis.

### Practitioner and patient retention

Retention will be facilitated through the practitioner and patient remuneration for each of the separate study procedures. *Patients* will be eligible for remuneration once they complete the mHealth questionnaire on day 23. The total remuneration amount will depend on the number of mHealth questionnaires completed. *Practitioners* will be eligible for remuneration at the end of the observational study phase after completing the UTAUT questionnaire. In addition, those who participate in the debriefing/interview session will receive additional remuneration.

### Training of providers and staff

Training for the participating Network staff and providers is executed using a “train the trainer” model. The study team will train Network Coordinators who in turn train the participating practitioners. Training of the Coordinators is done online using slides, a training guide, and a short guide. A series of training videos about using FollowApp.Care and the EDC are also made available to Coordinators and Practitioners. Training included the following: (1) general information about the study (i.e., contact information, study overview, structure, and use of the platform) and (2) specific information (i.e., recruitment, informed consent, data collection, and use of the Network EDC for data entry). Competency is assessed by the use of the FollowApp.Care platform and entering data in the database using mock participants. See Table 4 for an overview of the development process and the training tools.

**Table 4:** Overview of the training development process and training tools

Training Development Process	Training Tools
Draft by research team	Slides and videos
Cascading review by Coordinators	Quick Guide
Review by NDPBRN Executive Committee	Training Manual
Review by NIH Program Officer	Screening & Enrollment log
Approval by NIH	Screening workflow

### Sample size

A minimum of 150 and up to 215 dental providers will be recruited, and each provider will be expected to enroll an average of 21 patients for a total of 3147. Assuming a 40% missing data rate among enrolled patients throughout the duration of the study, there will be 1888 patients remaining. This includes patient non-response rates as well as item non-response rates for the outcome of interest during the study period. Given a sample of 1888, the GLMM model for repeated measures will be able to detect a 20% reduction in pain intensity between procedure groups over time with 80.0% power. The minimum sample size was derived by the “longpower” sample package in the R statistical computing environment.

### Statistical analysis

Standard descriptive statistics will be used to describe both patient and practitioners’ characteristics using 5 data capture methods: 1) the SUS questionnaire, 2) the mHealth Questionnaire distributed on pre-specified days post procedure, 3) the UTAUT questionnaire, 4) the debriefing/interviews, 5) and the practitioner eCRF. Summary statistics such as means, medians, and ranges will be produced for all measured continuous variables. Frequencies and percent contributions will be computed for all categorical and ordinal variables. Graphical methods like X-charts will examine distributions over time and identify potential, influential time points. The balance of baseline characteristics and measures between groups will be compared using appropriate tests, including chi-squared tests, student t-tests, and Wilcoxon rank-sum tests.

A challenge for this non-randomized study is confounders – pre-existing variables that affect the outcomes and differ between the treatment groups. In order to reduce bias, potential confounders variables will be adjusted in regression models for the analysis. Once participants discontinue the study, they will not be contacted and will not be replaced as the power calculation has accounted for them. If the discontinuation rate is high, the study team PIs will discuss how to proceed with the Network and NIDCR. We will create a missing category for all missing data and assess the need for any imputation methods.

We will estimate the difference in intensity, pain interference, and patient satisfaction dependent on the treatment type. The relative risk (RR) will be the measure of association reported along with 95% confidence intervals. Pain, interference, and satisfaction at the end of the study will be compared among the treatment categories. To model the differential treatment (CDT Grouping) effect on patients’ pain intensity, interference, and satisfaction, on, we will use GLMMs using a Poisson link. Models will include time, treatment type, age, gender, race/ethnicity, medications, and no medication methods as fixed effects. In addition, dentist and Network regions will be included as random effects in the models to account for correlations within clusters.

A secondary analysis will be considered for additional evaluation of the FollowApp.Care platform by the practitioners including assessment of usability and evaluation of fidelity. Means and corresponding estimates of precision (e.g., standard deviations and 95% confidence intervals) and frequency distributions with percentage contributions will be used to report the distribution of each metric. In addition, we will conduct a confirmatory factor analysis (CFA) for multilevel data to test the reliability and construct validity.

All statistical analyses will be performed at the standard significance level (α = 0.05) using R and Stata Statistical Software release 15 for StataCorp LP.

## Discussion

In our pilot study (AHRQ 1U18HS026135), we successfully explored how an innovative mobile health intervention (FollowApp.Care®) could optimize the quality of acute post-op dental pain management by collecting PRO data from patients after dental procedures. However, it also taught us a number of implementation barriers. Some of these barriers are not new to those collecting PRO data, including difficulties with optimizing workflows and minimizing logistical burdens, further exacerbated by the expanded precautionary settings during COVID; data management in light of staff turnover; and staff training and providers [30, 31]. Barriers specific to implementing dental PROs using mHealth included making sure that providers see and act upon alerts timely; a hybrid approach to recruitment of patients due to the COVID environment; inaccurate phone numbers, and phone carrier contractual issues that prevented text messaging.

These barriers can substantially hamper the implementation of PROs by the general dentist, to the detriment of advancing patient-centered care in the dental arena. In the POPS (**P**ost-**O**perative **P**ain **S**tudy) trial (UH3DE029158, Understanding Pain after Dental Procedures), we seek to address the gap in knowledge by implementing an existing mHealth technology (FollowApp.Care) in the dental office setting. Through the FollowApp.Care platform, we will actively track pain and other complications following dental procedures. We expect to identify specific pain experiences associated with surgical dental procedures promptly and precisely. We will also collect information about pain management strategies and medications recommended by the practitioner and utilized by the patient. The results of this study will provide insight into pain and other postoperative complications experienced by patients through approximately 3 weeks following common dental procedures. We will also be able to gauge to what extent practitioners are interested in adopting mHealth technology. We will also learn how patients like to be engaged with text messaging to report post-operative pain and complications.

## Limitation

This observational study offers insight into the distribution of patient-reported outcomes, while also capturing information on strategies used by practitioners for pain management. However, a challenge for this non-randomized study is confounders – pre-existing variables that affect the outcomes and differ between the treatment groups. In order to reduce bias, potential confounders variables will be adjusted for in regression models for the analysis. We will create a missing category for all missing data and assess the need for any imputation methods.

## Supplementary Information

**Additional file 1.** Appendix 1.

**Additional file 2.** Appendix 2.

**Additional file 3.** Appendix 3.

## Data Availability

Not applicable.

## Abbreviations

ADA: American Dental Association (ADA)
AAOMS: American Association of Oral and Maxillofacial Surgeons
AAE: American Association of Endodontists
API: Application program interface
APS-POQ-R: American Pain Society Patient Outcome Questionnaire
CDT: Code on Dental Procedures and Nomenclature (CDT Code)
CFA: Confirmatory factor analysis
cIRB: Central IRB
EDC: Electronic data capture system
GCP: Good clinical practice
GLMM: Generalized linear mixed model
HIT: Health information technology
IT: Information Technology
IR: Immediate-release
IRB: Institutional Review Board
mHealth: Mobile health
MOP: Manual of Procedures
PROs: Patient-reported outcomes (PROs)
OMFS: Oral and Maxillofacial Surgery
PROMIS: Patient RepoPatient-Reportedasurement Information System
RR: Relative Risk
SUS: System Usability Score
UAB: University of Alabama
US: United States
