# Supplementary material for: Study protocol: understanding pain after dental procedures, an observational study within the National Dental PBRN
Source: BMC Oral Health. 2022 Dec 9;22:581. doi: 10.1186/s12903-022-02573-9 (PMC9733211; doi:10.1186/s12903-022-02573-9)
Supplement: Supplementary file 1 — Additional file 1. Appendix 1. [file 12903_2022_2573_MOESM1_ESM.pdf]

A) Questions per day - NOT sorted by the order in which they are asked (for questions in ord

-----> Blue colored cells = question is asked in that Day's survey

-----> Light blue colored cells = question is asked in that Day's survey BUT not in that order (see Table B)

	Question	Day 0	Day 1	Day 3	Day 5	Day 7	Day 14	Day 21
**1**	How intense was your pain at its worst 24 hours before your procedure?							
**2**	What is your gender?							
**3**	What is your date of birth?							
**4**	Are you of Hispanic or Latino origin?							
**5**	What racial categories best describe you?							
**6**	What type of dental insurance do you have?							
**7**	Indicate your highest level of formal education							
**8**	How would you describe the neighborhood where you live?							
**9**	What is the ZIP Code where you live?							
**10**	Including you, how many people live in your household?							
**11**	What is your family's current annual household income from all sources?							
**12**	Good morning, thanks for participating in your dentist's study. Please click							
**13**	What is your level of pain right now?							
**14**	How intense was your pain at its worst following your procedure?							
**15**	***Is there any discomfort from the neighboring teeth or area close to***		*	*	*	*	*	*
**16**	***What medications have you taken related to your dental procedure in***		*	*	*	*	*	*
**17**	***If other, please list the medications you are taking:***		*	*	*	*	*	*
**18**	***How often have you taken pain medications related to your dental***		*	*	*	*	*	*
**19**	Does your bleeding seem better, about the same, or worse compared to							
**20**	Does your swelling seem better, about the same, or worse compared to							
**21**	Have you found it uncomfortable to eat any foods because of the pain from							
**22**	Have you had trouble pronouncing any words because of the pain from your dental procedure?							
**23**	Please select the one number below that best describes how much pain interfered or prevented you from:							
**24**	Doing activities out of bed such as walking sitting in a chair standing at the sink							
**25**	Falling asleep							
**26**	Staying asleep							
**27**	Were you allowed to participate in decisions about your pain treatment as much as you wanted to?							
**28**	Select the one number that best shows how satisfied you are with your pain treatment							
**29**	Did you use any non-medicine methods to relieve your pain?							
**30**	If other, please list the non-medicine method you used:							
**31**	Do you have any questions or concerns for Dr. [doctorlastname] ?							
**32**	Please write down your questions or concerns for Dr {doctorlastname} and							
**33**	I think that I would like to use this system frequently.							
**34**	I found the system unnecessarily complex.							
**35**	I thought the system was easy to use.							

36	I think that I would need the support of a technical person to be able to use
37	I found the various functions in this system were well integrated.
38	I thought there was too much inconsistency in this system.
39	I would imagine that most people would learn to use this system very
40	I found the system very cumbersome to use.
41	I felt very confident using the system.
42	I needed to learn a lot of things before I could get going with this system.
43	Ending screen

B) Questions per day - sorted by the order in which they are asked

	**Question**	**Day 0**	**Day 1**	**Day 3**	**Day 5**	**Day 7**	**Day 14**	**Day 21**
1	What is your sex/gender?							
2	In what year were you born?							
3	Are you of Hispanic or Latino origin?							
4	What racial categories best describe you?							
5	What type of dental insurance do you have? (Check all that apply)							
6	Indicate your highest level of formal education							
7	How would you describe the community where you live?							
8	What is the ZIP Code where you live?							
9	Including you, how many people live in your household?							
10	What is your family's current annual household income from all sources?							
11	How intense was your pain at its worst 24 hours before your procedure?							
12	Good morning, thanks for participating in your dentist's study. Please click							
13	What is your level of pain right now?							
14	How intense was your pain at its worst following your procedure?							
15	***Is there any discomfort from the neighboring teeth or area close to***		*	*	*	*	*	*
16	***What medications have you taken related to your dental procedure in***		*	*	*	*	*	*
17	***If other, please list the medications you are taking:***		*	*	*	*	*	*
18	***How often have you taken pain medications related to your dental***		*	*	*	*	*	*
19	Does your bleeding seem better, about the same, or worse compared to							
20	Does your swelling seem better, about the same, or worse compared to							
21	Have you found it uncomfortable to eat any foods because of the pain from							
22	Have you had trouble pronouncing any words because of the pain from your dental procedure?							
23	Please select the one number below that best describes how much pain interfered or prevented you from:							

24	Doing activities out of bed such as walking sitting in a chair standing at the sink						
25	Falling asleep						
26	Staying asleep						
15	***Is there any discomfort from the neighboring teeth or area close to***	*	*	*	*	*	*
27	Were you allowed to participate in decisions about your pain treatment as much as you wanted to?						
28	Select the one number that best shows how satisfied you are with your pain treatment						
29	Did you use any non-medicine methods to relieve your pain?						
30	If other, please list the non-medicine method you used:						
16	***What medications have you taken related to your dental procedure in***	*	*	*	*	*	*
17	***If other, please list the medications you are taking:***	*	*	*	*	*	*
18	***How often have you taken pain medications related to your dental***	*	*	*	*	*	*
31	Do you have any questions or concerns for Dr. [doctorlastname] ?						
32	Please write down your questions or concerns for Dr {doctorlastname} and						
33	I think that I would like to use this system frequently.						
34	I found the system unnecessarily complex.						
35	I thought the system was easy to use.						
36	I think that I would need the support of a technical person to be able to use						
37	I found the various functions in this system were well integrated.						
38	I thought there was too much inconsistency in this system.						
39	I would imagine that most people would learn to use this system very						
40	I found the system very cumbersome to use.						
41	I felt very confident using the system.						
42	I needed to learn a lot of things before I could get going with this system.						
43	Ending screen						

er, see Table B)	
Day 23	Notes
	*This question is asked in Days 1-21, but in different order. In Days 1-5, the question is asked
	*This question is asked in Days 1-21, but in different order. In Days 1-7, the question is asked
	*This question is asked in Days 1-21, but in different order. In Days 1-7, the question is asked
	*This question is asked in Days 1-21, but in different order. In Days 1-7, the question is asked

Day 23	Notes

**This question is asked in Days 1-21, but*
**This question is asked in Days 1-21, but*
**This question is asked in Days 1-21, but*
**This question is asked in Days 1-21, but*

This question is asked in Days 1-21, but

This question is asked in Days 1-21, but
This question is asked in Days 1-21, but
This question is asked in Days 1-21, but

earlier, in Days 7-21 is asked later in the survey
earlier, in Days 14-21 is asked later in the survey
earlier, in Days 14-21 is asked later in the survey
earlier, in Days 14-21 is asked later in the survey
