# Supplementary material for: Study protocol: understanding pain after dental procedures, an observational study within the National Dental PBRN
Source: BMC Oral Health. 2022 Dec 9;22:581. doi: 10.1186/s12903-022-02573-9 (PMC9733211; doi:10.1186/s12903-022-02573-9)
Supplement: Supplementary file 2 — Additional file 2. Appendix 2. [file 12903_2022_2573_MOESM2_ESM.docx]

**The System Usability Scale**

When a SUS is used, participants are asked to score the following 10 items with one of five responses that range from Strongly Agree to Strongly disagree:

1. I think that I would like to use this system frequently.
2. I found the system unnecessarily complex.
3. I thought the system was easy to use.
4. I think that I would need the support of a technical person to be able to use this system.
5. I found the various functions in this system were well integrated.
6. I thought there was too much inconsistency in this system.
7. I would imagine that most people would learn to use this system very quickly.
8. I found the system very cumbersome to use.
9. I felt very confident using the system.
10. I needed to learn a lot of things before I could get going with this system.
