# Supplementary material for: Study protocol: understanding pain after dental procedures, an observational study within the National Dental PBRN
Source: BMC Oral Health. 2022 Dec 9;22:581. doi: 10.1186/s12903-022-02573-9 (PMC9733211; doi:10.1186/s12903-022-02573-9)
Supplement: Supplementary file 3 — Additional file 3. Appendix 3. [file 12903_2022_2573_MOESM3_ESM.pdf]

Understanding Pain after Dental Procedures - POPS

NIDCR Protocol Number: 19-073-E

NIDCR Grant Number: 1 UG3 DE029158-01

Principal Investigator: Muhammad Walji, PhD

NIDCR Program Official: Dena Fischer, DDS, MSD, MS

NIDCR Medical Monitor: Kevin McBryde, MD

POPS Study Practitioner Questionnaires

Version Number: 3.0

Mar 29, 2022

1. eCRF Day 0 (Baseline Data to be collected on the day of the procedure)

Provider Identifier (autogenerated by NCC data capture system)
Did you receive a message from FollowApp.Care that this patient is enrolled? ☐Yes ☐No<br>If Yes: On what date did you receive this message: MM/DD/YYYY
What procedure(s) did you perform?<br><br>1) Endodontics - Surgery<br>a) Site (tooth #, quadrant, etc.) - indicate on odontogram<br>b) CDT (click all that apply):<br>i) D3410: apicoectomy - anterior<br>ii) D3421: apicoectomy - premolar (first root)<br>iii) D3425: Apicoectomy - molar (first root)<br>iv) D3426: Apicoectomy (each additional root)<br>v) D3428: bone graft in conjunction with periradicular surgery – per tooth, single site<br>vi) D3429: bone graft in conjunction with periradicular surgery – each additional contiguous tooth in the same surgical site<br>vii) D3430: retrograde filling - per root<br>viii) D3431: biologic materials to aid in soft and osseous tissue regeneration in conjunction with periradicular surgery<br>ix) D3432: guided tissue regeneration, resorbable barrier, per site, in conjunction with periradicular surgery<br>x) D3450: root amputation – per root<br>xi) D3460: endodontic endosseous implant<br>xii) D3470: intentional re-implantation (including necessary splinting)<br>xiii) D3910: surgical procedure for isolation of tooth with rubber dam<br>xiv) D3920: hemisection (including any root removal), not including root canal therapy<br>xv) D3950: canal preparation and fitting of preformed dowel or post<br>xvi) D3999: unspecified endodontic procedure, by report<br>c) Date: MM/DD/YYYY<br>d) Related diagnoses (click all that apply)<br>i) Symptomatic reversible pulpitis<br>ii) Symptomatic irreversible pulpitis<br>iii) Necrosis of the pulp<br>iv) Previously completed endodontic therapy

v) Acute apical abscess
vi) Chronic apical abscess
vii) Internal resorption of tooth
viii) External resorption of tooth
ix) Symptomatic periapical periodontitis
x) Asymptomatic periapical periodontitis
xi) Obliteration of root canal due to abnormal mineralization of tooth
xii) Other: Add text

2) Endodontics - Pulp Treatment (e.g. Pulpotomy, Pulpectomy)
   a) Site (tooth #, quadrant, etc.) – indicate on odontogram
   b) CDT (click all that apply):
      i) D3220: therapeutic pulpotomy (excluding final restoration) – removal of pulp coronal to the dentinocemental junction and application of medicament
      ii) D3221: pulpal debridement, primary and permanent teeth
      iii) D3222: partial pulpotomy for apexogenesis – permanent tooth with incomplete root development
      iv) D3230: pulpal therapy (resorbable filling) – anterior, primary tooth (excluding final restoration)
      v) D3240: pulpal therapy (resorbable filling) – posterior, primary tooth (excluding final restoration)
   c) Date: MM/DD /YYYY
   d) Related diagnoses
      i) Symptomatic reversible pulpitis
      ii) Symptomatic irreversible pulpitis
      iii) Necrosis of the pulp
      iv) Previously completed endodontic therapy
      v) Acute apical abscess
      vi) Chronic apical abscess
      vii) Internal resorption of tooth
      viii) External resorption of tooth
      ix) Symptomatic periapical periodontitis
      x) Asymptomatic periapical periodontitis
      xi) Obliteration of root canal due to abnormal mineralization of tooth
      xii) Tooth fracture with pulp involvement
      xiii) Other: Add text

3) Endodontics – Non-Surgical (e.g. RCT, Re-treat, Apexification)
   a) Site (tooth #, quadrant, etc.) – indicate on odontogram
   b) CDT (click all that apply):
      i) D3310: endodontic therapy, anterior tooth (excluding final restoration)

ii) D3320: endodontic therapy, premolar tooth (excluding final restoration)
iii) D3330: endodontic therapy, molar tooth (excluding final restoration)
iv) D3331: treatment of root canal obstruction; non-surgical access
v) D3332: incomplete endodontic therapy; inoperable, unrestorable or fractured tooth
vi) D3333: internal root repair of perforation defects
vii) D3346: retreatment of previous root canal therapy – anterior
viii) D3347: retreatment of previous root canal therapy – premolar
ix) D3348: retreatment of previous root canal therapy – molar
x) D3351: apexification/recalcification – initial visit (apical closure/calcific repair of perforations, root resorption, etc.)
xi) D3352: apexification/recalcification – interim medication replacement
xii) D3353: apexification/recalcification – final visit (includes completed root canal therapy – apical closure/calcific repair of perforations, root resorption, etc.)

c) Date: MM/DD /YYYY
d) Related diagnoses
i) Symptomatic reversible pulpitis
ii) Symptomatic irreversible pulpitis
iii) Necrosis of the pulp
iv) Previously completed endodontic therapy
v) Acute apical abscess
vi) Chronic apical abscess
vii) Internal resorption of tooth
viii) External resorption of tooth
ix) Symptomatic periapical periodontitis
x) Asymptomatic periapical periodontitis)
xi) Obliteration of root canal due to abnormal mineralization of tooth
xii) Other: Add text

4) Perio - Surgical
a) Site (tooth #, quadrant, etc.) – indicate on odontogram
b) CDT (click all that apply):
i. D4210: gingivectomy or gingivoplasty – four or more contiguous teeth or tooth bounded spaces per quadrant
ii. D4211: gingivectomy or gingivoplasty – one to three contiguous teeth or tooth bounded spaces per quadrant
iii. D4212: gingivectomy or gingivoplasty to allow access for restorative procedure, per tooth
iv. D4230: anatomical crown exposure – four or more contiguous teeth or bounded tooth spaces per quadrant
v. D4231: anatomical crown exposure – one to three teeth or bounded

tooth spaces per quadrant

vi. D4240: gingival flap procedure, including root planing – four or more contiguous teeth or tooth bounded spaces per quadrant

vii. D4241: gingival flap procedure, including root planing – one to three contiguous teeth or tooth bounded spaces per quadrant

viii. D4245: apically positioned flap

ix. D4249: clinical crown lengthening – hard tissue

x. D4260: osseous surgery (including elevation of a full thickness flap and closure) – four or more contiguous teeth or tooth bounded spaces per quadrant

xi. D4261: osseous surgery (including elevation of a full thickness flap and closure) – one to three contiguous teeth or tooth bounded spaces per quadrant

xii. D4263: bone replacement graft – retained natural tooth – first site

xiii. in quadrant

xiv. D4264: bone replacement graft – retained natural tooth – each additional site in quadrant

xv. D4266: guided tissue regeneration – resorbable barrier, per site

xvi. D4267: guided tissue regeneration – non-resorbable barrier, per site (includes membrane removal)

xvii. D4268: surgical revision procedure, per tooth

xviii. D4270: pedicle soft tissue graft procedure

xix. D4273: autogenous connective tissue graft procedure (including donor and recipient surgical sites) first tooth, implant or edentulous tooth position in graft

xx. D4275: non-autogenous connective tissue graft (including recipient site and donor material) first tooth, implant, or edentulous tooth position in graft

xxi. D4276: combined connective tissue and double pedicle graft, per tooth

xxii. D4277: free soft tissue graft procedure (including recipient and donor surgical sites) first tooth, implant, or edentulous tooth position in graft

xxiii. D4278: free soft tissue graft procedure (including recipient and donor surgical sites) each additional contiguous tooth, implant, or edentulous tooth position in same graft site

xxiv. D4283: autogenous connective tissue graft procedure (including donor and recipient surgical sites) – each additional contiguous tooth, implant or edentulous tooth position in same graft site

xxv. D4285: non-autogenous connective tissue graft procedure (including recipient surgical site and donor material) – each additional contiguous tooth, implant or edentulous tooth position in same graft site

c) Date: MM/DD/YYYY

d) Related diagnoses

- i) Localized Periodontitis
- ii) Generalized Periodontitis
- iii) Gingival abscess
- iv) Periodontal abscess
- v) Periodontal and endodontic lesion
- vi) Insufficient clinical crown height
- vii) Dental peri-implant mucositis
- viii) Dental peri-implantitis
- ix) Other: Add text

5) Extractions - Simple
   a) Site (tooth #, quadrant, etc.) – indicate on odontogram
   b) CDT (click all that apply):
      i) D7111: extraction, coronal remnants – primary tooth
      ii) D7140: extraction, erupted tooth or exposed root (elevation and/or forceps removal)
   c) Date: MM/DD/YYYY
   d) Related diagnoses
      i) Abnormalities of size and form of teeth
      ii) Tooth crowding
      iii) Tooth eruption disorder
      iv) Non-restorable carious tooth
      v) Periodontitis
      vi) Fracture of tooth
      vii) Pericoronitis
      viii) Other: Add text

6) Extractions - Surgical
   a) Site (tooth #, quadrant, etc.) – indicate on odontogram
   b) CDT (click all that apply):
      i) D7210: extraction, erupted tooth requiring removal of bone and/or sectioning of tooth, and including elevation of mucoperiosteal flap if indicated
      ii) D7220: removal of impacted tooth – soft tissue
      iii) D7230: removal of impacted tooth – partially bony
      iv) D7240: removal of impacted tooth – completely bony
      v) D7241: removal of impacted tooth – completely bony, with unusual surgical complications
      vi) D7250: removal of residual tooth roots (cutting procedure)
      vii) D7251: coronectomy – intentional partial tooth removal
   c) Date: MM/DD/YYYY
   d) Related diagnoses
      i) Retained dental root

- ii) Non-restorable carious tooth
- iii) Pulp necrosis
- iv) Symptomatic periapical periodontitis
- v) Chronic apical abscess
- vi) Tooth crowding
- vii) Fracture of tooth
- viii) Impacted tooth
- ix) Pericoronitis
- x) Other: Add text

7) Implants - Surgical Procedures
   a) Site (tooth #, quadrant, etc.) – indicate on odontogram
   b) CDT (click all that apply):
      i) D6010: surgical placement of implant body: endosteal implant
      ii) D6011: surgical access to an implant body (second stage implant surgery)
      iii) D6012: surgical placement of interim implant body for transitional prosthesis: endosteal implant
      iv) D6013: surgical placement of mini implant
      v) D6040: surgical placement: eposteal implant
      vi) D6050: surgical placement: transosteal implant
      vii) D6100: implant removal, by report
      viii) D6101: debridement of a peri-implant defect or defects surrounding a single implant, and surface cleaning of the exposed implant surfaces, including flap entry and closure
      ix) D6102: debridement and osseous contouring of a peri-implant defect or defects surrounding a single implant and includes surface cleaning of the exposed implant surfaces, including flap entry and closure
      x) D6103: bone graft for repair of peri-implant defect – does not include flap entry and closure
      xi) D6104: bone graft at time of implant placement
      xii) D6081: scaling and debridement in the presence of inflammation or mucositis of a single implant, including cleaning of the implant surfaces, without flap entry and closure
   c) Date: MM/DD/YYYY
   d) Related diagnoses
      i) Dental peri-implant mucositis
      ii) Dental peri-implantitis
      iii) Failure of osseointegration of dental implant
      iv) Acquired absence of teeth
      v) Other: Add text

8) Oral Surgery Other

a) Site (tooth #, quadrant, etc.) – indicate on odontogram
b) CDT (click all that apply):
   i) D7260: oroantral fistula closure
   ii) D7261: primary closure of a sinus perforation
   iii) D7270: tooth re-implantation and/or stabilization of accidentally evulsed or displaced tooth
   iv) D7272: tooth transplantation (includes re-implantation from one site
   v) to another and splinting and/or stabilization)
   vi) D7280: exposure of an unerupted tooth
   vii) D7282: mobilization of erupted or malpositioned tooth to aid eruption
   viii) D7283: placement of device to facilitate eruption of impacted tooth
   ix) D7285: incisional biopsy of oral tissue – hard (bone, tooth)
   x) D7286: incisional biopsy of oral tissue – soft
   xi) D7290: surgical repositioning of teeth
   xii) D7291: transseptal fiberotomy/supra crestal fiberotomy, by report
   xiii) D7292: placement of temporary anchorage device [screw retained plate] requiring flap
   xiv) D7298: removal of temporary anchorage device [screw retained plate], requiring flap
   xv) D7293: placement of temporary anchorage device requiring flap
   xvi) D7299: removal of temporary anchorage device, requiring flap
   xvii) D7294: placement of temporary anchorage device without flap; includes device removal
   xviii) D7300: removal of temporary anchorage device without flap
   xix) D7295: harvest of bone for use in autogenous grafting procedure
   xx) D7296: corticotomy – one to three teeth or tooth spaces, per quadrant
   xxi) D7297: corticotomy – four or more teeth or tooth spaces, per quadrant
   xxii) D7310: alveoloplasty in conjunction with extractions – four or more teeth or tooth spaces, per quadrant
   xxiii) D7311: alveoloplasty in conjunction with extractions – one to three teeth or tooth spaces, per quadrant
   xxiv) D7320: alveoloplasty not in conjunction with extractions – four or more teeth or tooth spaces, per quadrant
   xxv) D7321: alveoloplasty not in conjunction with extractions – one to three teeth or tooth spaces, per quadrant
   xxvi) D7340: vestibuloplasty – ridge extension (secondary epithelialization)
   xxvii) D7350: vestibuloplasty – ridge extension (including soft tissue grafts, muscle reattachment, revision of soft tissue attachment and management of hypertrophied and hyperplastic tissue)
   xxviii) D7410: excision of benign lesion up to 1.25 cm
   xxix) D7411: excision of benign lesion greater than 1.25 cm
   xxx) D7412: excision of benign lesion, complicated

xxxi) D7413: excision of malignant lesion up to 1.25 cm
xxxii) D7414: excision of malignant lesion greater than 1.25 cm
xxxiii) D7415: excision of malignant lesion, complicated
xxxiv) D7440: excision of malignant tumor – lesion diameter up to 1.25 cm
xxxv) D7441: excision of malignant tumor – lesion diameter greater than 1.25 cm
xxxvi) D7450: removal of benign odontogenic cyst or tumor – lesion diameter up to 1.25 cm
xxxvii) D7451: removal of benign odontogenic cyst or tumor – lesion diameter greater than 1.25 cm
xxxviii) D7460: removal of benign nonodontogenic cyst or tumor – lesion diameter up to 1.25 cm
xxxix) D7461: removal of benign nonodontogenic cyst or tumor – lesion diameter greater than 1.25 cm
xl) D7465: destruction of lesion(s) by physical or chemical method, by report
xli) D7471: removal of lateral exostosis (maxilla or mandible)
xlii) D7472: removal of torus palatinus
xliii) D7473: removal of torus mandibularis
xliv) D7485: reduction of osseous tuberosity
xlv) D7490: radical resection of maxilla or mandible
xlvi) D7510: incision and drainage of abscess – intraoral soft tissue
xlvii) D7511: incision and drainage of abscess – intraoral soft tissue – complicated (includes drainage of multiple fascial spaces)
xlviii) D7520: incision and drainage of abscess – extraoral soft tissue
xlix) D7521: incision and drainage of abscess – extraoral soft tissue –
l) complicated (includes drainage of multiple fascial spaces)
li) D7530: removal of foreign body from mucosa, skin, or subcutaneous alveolar tissue
lii) D7540: removal of reaction producing foreign bodies, musculoskeletal system
liii) D7550: partial ostectomy/sequestrectomy for removal of non-vital bone
liv) D7610: maxilla – open reduction (teeth immobilized, if present)
lv) D7620: maxilla – closed reduction (teeth immobilized, if present)
lvi) D7630: mandible – open reduction (teeth immobilized, if present)
lvii) D7640: mandible – closed reduction (teeth immobilized, if present)
lviii) D7650: malar and/or zygomatic arch – open reduction
lix) D7660: malar and/or zygomatic arch – closed reduction
lx) D7670: alveolus – closed reduction, may include stabilization of teeth
lxi) D7671: alveolus – open reduction, may include stabilization of teeth
lxii) D7680: facial bones – complicated reduction with fixation and multiple surgical approaches
lxiii) D7710: maxilla – open reduction
lxiv) D7720: maxilla – closed reduction D7730 mandible – open reduction
lxv) D7740: mandible – closed reduction

lxvi) D7750: malar and/or zygomatic arch – open reduction
lxvii) D7760: malar and/or zygomatic arch – closed reduction
lxviii) D7770: alveolus – open reduction stabilization of teeth
lxix) D7771: alveolus, closed reduction stabilization of teeth
lxx) D7780: facial bones – complicated reduction with fixation and multiple approaches
lxxi) D7810: open reduction of dislocation
lxxii) D7820: closed reduction of dislocation
lxxiii) D7830: manipulation under anesthesia
lxxiv) D7840: condylectomy
lxxv) D7850: surgical discectomy, with/without implant
lxxvi) D7852: disc repair
lxxvii) D7854: synovectomy
lxxviii) D7856: myotomy
lxxix) D7858: joint reconstruction
lxxx) D7860: arthrotomy
lxxxi) D7865: arthroplasty
lxxxii) D7870: arthrocentesis
lxxxiii) D7871: non-arthroscopic lysis and lavage
lxxxiv) D7872: arthroscopy – diagnosis, with or without biopsy
lxxxv) D7873: arthroscopy: lavage and lysis of adhesions
lxxxvi) D7874: arthroscopy: disc repositioning and stabilization
lxxxvii) D7875: arthroscopy: synovectomy
lxxxviii) D7876: arthroscopy: discectomy
lxxxix) D7877: arthroscopy: debridement
xc) D7910: suture of recent small wounds up to 5 cm
xci) D7911: complicated suture – up to 5 cm
xcii) D7912: complicated suture – greater than 5 cm
xciii) D7920: skin graft (identify defect covered, location and type of graft)
xciv) D7922: placement of intra-socket biological dressing to aid in hemostasis or clot stab
xcv) D7941: osteotomy – mandibular rami
xcvi) D7943: osteotomy – mandibuilization, per site
xcvii) D7944: osteotomy – segmented or subapical
xcviii) D7945: osteotomy – body of mandible
xcix) D7946: LeFort I (maxilla – total)
c) D7947: LeFort I (maxilla – segmented)
ci) D7948: LeFort II or LeFort III (osteoplasty of facial bones for midface hypoplasia or retrusion) – without bone graft
cii) D7949: LeFort II or LeFort III – with bone graft
ciii) D7950: osseous, osteoperiosteal, or cartilage graft of the mandible or maxilla – autogenous or nonautogenous, by report

civ) D7951: sinus augmentation with bone or bone substitutes via a lateral open approach
cv) D795: sinus augmentation via a vertical approach
cvi) D7953: bone replacement graft for ridge preservation – per site
cvii) D7955: repair of maxillofacial soft and/or hard tissue defect
cviii) D7961: buccal / labial frenectomy (frenulectomy)
cix) D7962: lingual frenectomy (frenulectomy)
cx) D7963: surgical placement of craniofacial implant – extra oral
cxi) D7970: excision of hyperplastic tissue – per arch
cxii) D7971: excision of pericoronal gingiva
cxiii) DD7972: surgical reduction of fibrous tuberosity
cxiv) D7979: non – surgical sialolithotomy
cxv) D7980: surgical sialolithotomy
cxvi) D7981: excision of salivary gland, by report
cxvii) D7982: sialodochoplasty
cxviii) D7983: closure of salivary fistula
cxix) D7990: emergency tracheotomy
cxx) D7991: coronoidectomy
cxxi) D7993: surgical placement of craniofacial implant – extra oral
cxxii) D7994: surgical placement: zygomatic implant
cxxiii) D7995: synthetic graft – mandible or facial bones, by report
cxxiv) D7996: implant-mandible for augmentation purposes (excluding alveolar ridge), by report
cxxv) D7997: appliance removal (not by dentist who placed appliance), includes removal of archbar
cxxvi) D7998: intraoral placement of a fixation device not in conjunction with a fracture
cxxvii) D7999: unspecified oral surgery procedure, by report

c) Date: MM/DD/YYYY
d) Related diagnoses
   i) Fibrous epulis
   ii) Flabby alveolar ridge
   iii) Insufficient biological width
   iv) Exostosis of jaw
   v) Irregular alveolar process
   vi) Sequestrum of jawbone
   vii) Impacted tooth
   viii) Other: Add text

As part of your pain management plan, what medications were prescribed?
(Select all that apply - checkboxes)

- None

- Acetaminophen (Tylenol)
  - Name
  - Dose
  - Frequency
- Ibuprofen (Advil, Motrin)
  - Name
  - Dose
  - Frequency
- Acetaminophen and Ibuprofen (Advil Dual Action)
  - Name
  - Dose
  - Frequency
- Naproxen (Aleve)
  - Name
  - Dose
  - Frequency
- Opioids (Codeine, Hydrocodone, Oxycodone)
  - Name
  - Dose
  - Frequency
- Combination Drug (Tylenol #3, Norco, Vicodin, Percocet, Percodan, Vicoprofen)
  - Name
  - Dose
  - Frequency
- Antibiotics (Amoxycillin, Penicillin, Clindamycin, Erythromycin)
  - Name
  - Dose
  - Frequency
- Steroid (Medrol)
  - Name
  - Dose
  - Frequency
- Other medications for dental pain
  - Name
  - Dose
  - Frequency

Did the patient receive a long-acting local anesthetic as part of the procedure? (bupivacaine, etidocaine)

- Yes
- No

What do you expect the patient's pain level to be?<br><br>• 0 - 10 scale<br>o No pain (0) - Moderate pain (5) - Worst imaginable pain (10)<br><br>a. Immediate post-op (Day 1)<br>a. Short-term (Day 3 - 5)<br>b. Long -term (after the 1st week)
On what day post-procedure do you anticipate the patient will be free of pain?<br><br>Day 1-21

2. eCRF Day 21 – Data to be completed 21 days after visit

Did the patient contact your office regarding their procedure within 21 days after the procedure?<br>If so, was it via:<br>• No contact<br>• Phone call<br>• Email<br>• Office visit<br>o Unscheduled<br>o Scheduled
Did you change your pain management plan?<br>[Display Day 0 Plan]<br>• No<br>• Yes (Click all that apply)<br>o Due to information received through the app<br>o Due to office communication (phone call, email, office visit)<br>o Other<br><br>If Yes, what were the changes (select all that apply.):<br>• Stop medications<br>• Increase medication dosage/frequency<br>• Decrease medication dosage/frequency<br>• Switch medications

o Name<br>o Dose<br>o Frequency<br>• Add additional medications<br>o Name<br>o Dose<br>o Frequency<br>• Other<br>o Free text

3. UTAUT Questionnaire for Practitioners after the study has been completed (via REDCap)

Please rate your agreement with each statement below (Select from 1 to 7, from Strongly Disagree {1} to Strongly Agree {7})

1. I find FollowApp useful in my job.
2. Using FollowApp enables me to accomplish tasks more quickly.
3. Using FollowApp increases my productivity.
4. Using FollowApp will increase my chances of increasing my income
5. My interaction with FollowApp is clear and understandable.
6. It is easy for me to become skillful at using FollowApp.
7. FollowApp is easy to use.
8. Learning to operate FollowApp is easy for me.
9. Using FollowApp is a good idea.
10. FollowApp makes work more interesting.
11. Working with FollowApp is fun.
12. I like working with FollowApp.
13. People who influence my behavior think that I should use FollowApp.
14. People who are important to me think that I should use FollowApp.

15. The clinical management has been helpful in the use of FollowApp.
16. In general, my dental office has supported the use of FollowApp.
17. I have the resources necessary to use FollowApp.
18. I have the knowledge necessary to use FollowApp.
19. FollowApp is not compatible with other systems I use.
20. A specific person (or group) is available for assistance with FollowApp difficulties.
21. I could complete a job or task using FollowApp if there was no one around to tell me what to do as I go.
22. I could complete a job or task using FollowApp if I could call someone for help if I got stuck.
23. I could complete a job or task using FollowApp if I had a lot of time to complete the job for which the software was provided.
24. I could complete a job or task using FollowApp if I had just the built-in help functionality for assistance.
25. I feel apprehensive about using FollowApp.
26. It scares me to think that I could lose a lot of information using FollowApp by hitting the wrong key.
27. I hesitate to use FollowApp for fear of making mistakes I cannot correct.
28. FollowApp is somewhat intimidating to me.
29. If available, I intend to continue using FollowApp.
30. If available, I predict I would continue using FollowApp.
31. If available, I plan to continue using FollowApp.
